# Global health education in United States anesthesiology residency programs: a survey of resident opportunities and program director attitudes

**DOI:** 10.1186/s12909-017-1056-3

**Published:** 2017-11-16

**Authors:** Gunisha Kaur, Sheida Tabaie, Jasmit Brar, Virginia Tangel, Kane O. Pryor

**Affiliations:** 525 East 68th Street Box 124, New York, NY 10065 USA

**Keywords:** Residency education, Global health training, Anesthesiology education, International electives, Graduate medical education

## Abstract

**Background:**

Interest in global health during postgraduate residency training is increasing across medical specialties, and multiple disciplines have categorized global health training opportunities in their arena. No such cataloging exists for anesthesiology residency programs. The aim of this study was to assess and characterize global health opportunities and the attitudes of program directors (PDs) in U.S. anesthesiology residency programs towards this training.

**Methods:**

A cross-sectional 20-question survey on global health opportunities was distributed to 128 ACGME accredited anesthesiology residency program directors via email between October 2015 and January 2016. Descriptive statistics and exploratory inferential analyses were applied. Maximal nonresponse selection bias was estimated.

**Results:**

The overall response rate was 44%. Of those who responded, 61% reported that their residency program had a global health elective, with a maximal bias estimate of 6.5%. 45% of program directors with no global health elective reported wanting to offer one. 77% of electives have articulated educational goals, but there is substantial heterogeneity in curricula offered. Program director attitudes regarding the value of global health programs differed significantly between those with and without existing programs.

**Conclusions:**

The proportion of U.S. anesthesiology residency programs offering global health electives is similar to that in other medical specialties. There is inconsistency in program structure, goals, curriculum, and funding. Attitudes of program directors differ between programs with and without electives, which may reflect bidirectional influence to be investigated further. Further studies are needed to codify curricula, assess effectiveness, and validate methodologies.

**Electronic supplementary material:**

The online version of this article (10.1186/s12909-017-1056-3) contains supplementary material, which is available to authorized users.

## Background

Global health is a field dedicated to addressing medical problems that transcend national boundaries. Interest in global health during postgraduate residency training is increasing across medical specialties. Anesthesiology resident physicians have demonstrated their interest in global health work. A recent study of resident physicians showed an overwhelming 91% of 460 participants indicating their interest in global health opportunities; additionally, 78% of participants agreed that the availability of a global health outreach residency track would influence their program ranking during the residency match [1]. Global health electives during residency have been well described for other medical specialties such as internal medicine, pediatrics, general surgery, and obstetrics and gynecology [2]. Information on global health exposure and offerings among anesthesiology training programs has yet to be described.

As resident physicians engage in global health electives with appropriate training, associated benefits include improved medical knowledge and diagnostic skills, increased awareness of social determinants of health, enhanced cultural understanding, exposure to a broad spectrum of illnesses, and greater appreciation of resource utilization [3]. However, without appropriate education and preparation, medical work in the developing world may disrupt existing healthcare infrastructure, inappropriately utilize scarce resources, and compromise patient care due to substandard provision of care by trainees [4]. Despite the increase in global health electives and the potential benefits and challenges of these programs, current global health offerings, level of preparation and training provided, and attitudes of program directors towards such education is a virtually unexplored domain in anesthesiology.

The aim of this study was to assess and characterize current global health opportunities and the attitudes of program directors in U.S. anesthesiology residency programs towards this training.

## Methods

### Participants

A cross-sectional survey of the program directors of the 133 anesthesiology residency programs accredited by the Accreditation Council for Graduate Medical Education (ACGME) was conducted. Program directors were identified through the list of programs by specialty published on the public ACGME website.^1^ E-mail addresses were confirmed first directly through program websites, and if unavailable through these websites, by directly contacting the telephone number listed for the program online. Five programs were excluded from the study as a result of failed contact due to non-functional e-mail addresses.

### Survey conduct

Data were collected between October 2015 and January 2016. An initial invitation and two reminders were sent via e-mail over the course of the data collection period. These e-mails provided a link to an anonymous web-based survey hosted by the online survey company SurveyMonkey (Palo Alto, CA). The survey consisted of 20 questions (Additional file 1) designed to evaluate the existence of a global health elective, attitudes towards global health education generally, and, for directors of existing programs, the content and structure of their programs. IP addresses associated with survey response were not collected. Informed consent was received from all participants as part of the survey process, prior to the presentation of any questions.

### Statistical analyses

Because the study design aimed to sample the entire population of interest (i.e. all U.S. program directors), a priori power analysis and estimate of required sample size was not indicated. Descriptive statistics were applied, and Pearson’s chi-squared tests performed to assess differences in categorical outcomes. Missing data was handled by listwise deletion for each analysis. All analyses were conducted in Stata IC 13 (StataCorp, College Station, TX).

Because survey nonresponse could not be assumed to be completely independent from the variables measured in the survey (i.e. nonresponses were more likely to represent data missing not at random (MNAR) than data missing completely at random (MCAR)), two assessments of potential nonresponse selection bias were performed. First, an estimate for the maximal estimator bias using the technique derived by Bethlehem [5] (eq. 14) was applied. This method, used to estimate the bias resulting from self-selection and non-response in surveys, provides an upper bound for bias when the standard deviation of response probability is at its maximum possible value (based on population size), and it is assumed that there is maximal relationship between response behavior and the target variable. Second, a ‘continuum of resistance’ model was applied. The assumptions of this model are that nonrespondents most closely resemble respondents who respond only after substantial time lags or reminders; thus, the absence of a significant difference between early and late responses suggests a low nonresponse bias [6]. Responses were thus partitioned into subsets of ‘early’ and ‘late’ responses, and Pearson’s chi-squared tests used to assess differences in the prevalence of global health programs. Further, as MNAR nonresponse implies a nonprobability sample, confidence intervals are not presented for descriptive statistics.

Approval for this study was received from the IRB of Weill Cornell Medicine. The conduct of the study and manuscript adhere to the guidelines for reporting of observational studies, as outlined by the STROBE statement [7].

## Results

56 of 128 program directors responded to the study, an overall response rate of 44%. Of those who responded, 61% (*n* = 34) reported that their residency program had a global health elective, and 45% (*n* = 10) of program directors with no global health elective reported wanting to offer one. Thus, cumulatively, 79% (*n* = 44) of programs either presently offer or would like to offer a global health elective. Shown in Table 1, the most cited reasons for not having a global health program were a lack of funding (59%, *n* = 13), a lack of a global health partner or program through which to offer an elective (50%, *n* = 11), and a lack of time within the parameters of training (36%, *n* = 8) (Additional file 2).

**Table 1:** Survey responses from ACGME residency program directors who do not offer a global health elective

Sample without global health elective	Total
	*N* = 22
Would like residency training to have global health elective, no. (%)	10 (45.5)
Reasons for lack of global health elective	
Lack of interest	3 (13.6)
Lack of perceived volume of cases abroad	0 (0)
Lack of funding	13 (59.1)
Lack of time within parameters of training	8 (36.4)
Lack of global health partner/program through which to offer an elective	11 (50.0)

### Characteristics of existing programs

Table 2 shows the structural characteristics of existing global health electives. A variable number of residents per year were allowed to participate in global health electives, ranging from 1 to 18, with 12% (*n* = 4) of programs permitting more than 5 residents per year. 71% (*n* = 24) of global health electives offered opportunities for post-graduate year 3 residents to participate, while 94% (*n* = 32) provided opportunities for residents in post-graduate year 4. The median elective time was 10 days (range 5–30), and 97% (n = 32) of programs did not require residents to use any vacation time to complete the elective.

**Table 2:** Survey responses from ACGME residency program directors who offer a global health elective

Sample with global health electives	Total
	*N* = 34
Partners, no. (%)	*N* = 32
Internal department funding or endowment	12 (37.5)
Institutional funding	4 (12.5)
Nongovernmental organization funding	5 (15.6)
Grant funding not from a nongovernmental organization	3 (9.4)
Residents must find their own funding	1 (3.13)
Curriculum includes biosocial determinants of health, no. (%)	*N* = 34
Contains content on poverty	23 (67.6)
Contains content on access to natural resources	14 (41.2)
Contains content on discrimination	9 (26.5)
Contains information on gender violence	3 (8.8)
Goals of global health elective, no. (%)	*N* = 34
Programs with educational outcome goals	26 (76.5)
Programs with required research component	6 (17.6)

Seventy-seven percent (*n* = 26) of programs reported that their department had established educational outcome goals for each resident while away on a global health elective. 18% (*n* = 6) reported having a required research component. There was heterogeneity in the education curricula and training provided to residents. Beyond clinical preparation, while 68% (*n* = 23) of programs addressed poverty, other biosocially relevant topics were covered less frequently. 41% (*n* = 14) addressed access to natural resources, 26% (*n* = 9) addressed discrimination, and 9% (*n* = 3) addressed gender violence. In 61% (*n* = 19) of electives, residents were evaluated by intradepartmental attending anesthesiologists, while 19% (*n* = 6) were evaluated by attending anesthesiologists based in the international location.

Thirty-two program directors responded to a subset of questions on program funding. 38% (*n* = 12) of program directors reported that funding for their electives was derived from internal department sponsorship, while 16% (*n* = 5) received funds from non-government organizations, and 13% (*n* = 4) from their home institutions. 33% (*n* = 10) of programs with electives reported that they offer financial support to residents seeking non-program-sponsored mission trips abroad. A total of 33 unique countries were reported as sites of global health electives throughout Central and South America, Africa, and Asia. The countries in which most global health electives took place were China, the Dominican Republic, Ecuador, Ethiopia, and India.

### Attitudes toward Global Health electives

Table 3 shows an exploratory analysis of the attitudes of program directors toward global health electives. Responses to this subset of questions were received from 31 programs that offer electives, and 19 programs that do not. When questioned about the perceived benefits of global health electives, those with and without current programs had similar levels of agreement on the benefit of providing needed health care to underserved areas of developing countries (77% vs 79%, *P* = 0.90), and on the benefit of personal, professional, and institutional development in the spheres of service-oriented action, humanitarian contribution, and outreach to underprivileged individuals and societies (81% vs 74%, *P* = 0.56). In contrast, those with current programs were significantly more likely than those without programs to agree that benefits included advancing education in the field of global anesthesia (90% vs 42%, *P* < 0.001), generating effective and engaging programs in the developing world that give residents and faculty the opportunity to become well-rounded, globally conscious physicians (74% vs 42%, *P* = 0.02), the opportunity for residents to become leaders in the field of global anesthesia research and contribute to global health literature (68% vs 32%, *P* = 0.01), and developing cross-institutional collaborations (68% vs 32%, *P* = 0.01).

**Table 3:** Attitudes of program directors towards global health electives

	With program	Without program	Total	Comparisons
	*N* = 31	*N* = 19	*N* = 50	*P*
Sample characteristics				
Value of global health in anesthesiology residency, no. (% indicating strongly or somewhat agree)				
Electives are important in training of anesthesiology residents	27 (87.1)	8 (42.1)	35 (70.0)	< 0.001
Exposure to global health care is a valuable experience	29 (93.5)	13 (68.4)	42 (84.0)	0.02
Exposure to global health care should be required of anesthesiology residency training	10 (32.3)	1 (5.3)	11 (22.0)	0.03
Perceived benefits of global health electives, no. (%)				
Advancing education in the field of global anesthesia	28 (90.3)	8 (42.1)	36 (72.0)	< 0.001
Generating effective and engaging programs in the developing world that give my department ‘s residents and faculty the opportunity to become well-rounded, globally conscious physicians	23 (74.2)	8 (42.1)	31 (62.0)	0.02
The opportunity for residents to become leaders in the field of global anesthesia research and contribute to global health literature	21 (67.7)	6 (31.6)	27 (54.0)	0.01
Developing cross-institutional collaborations	21 (67.7)	6 (31.6)	27 (54.0)	0.01
Personal, professional, and institutional development in the spheres of service-oriented action, humanitarian contribution, and outreach to underprivileged individuals and societies	25 (80.6)	14 (73.7)	39 (78.0)	0.56
Providing needed health care to underserved area of developing countries	24 (77.4)	15 (78.9)	39 (78.0)	0.90

For a subset of questions on the perceived value of global health electives, respondents were able to indicate whether they strongly agreed, somewhat agreed, somewhat disagreed, strongly disagreed, or had no opinion. Those with programs were significantly more likely to say that they strongly or somewhat agreed that exposure to global health care is a valuable experience for anesthesiology residents (94% vs 68%, *P* = 0.02), and is important for the training of anesthesiology residents (87% vs 42%, *P* < 0.001). Those with programs were also significantly more likely to say that exposure to global health electives should be a required component of residency (32% vs 5%, *P* = 0.03).

### Assessment of bias

The initial email to program directors was sent on November 10, 2015, with follow-up emails on December 1, 2015 and January 23, 2016. There was no significant difference in global health elective status between early and late respondents when the partition was set at December 1, 2015 (early: *n* = 17, *P* = 0.69), nor when it was set at January 1, 2016 (early: *n* = 37, *P* = 0.79). The estimate for the absolute value of maximum bias using the method derived by Bethlehem was 6.5%.

## Discussion

This study assessed current global health opportunities in anesthesiology residency programs by a survey of ACGME accredited anesthesiology residency program directors (PDs). Our survey reports that 61% of responding programs have global health electives. The calculated maximum bias in this estimate was 6.5%, supporting the generalizability of our results. This proportion is on par with residency global health opportunities in other medical specialties, where 71% of emergency medicine [8], 61% of orthopedic surgery [9], 57% of internal medicine [10], 52% of pediatric [11], 74% of family medicine [12], and 33% of general surgery programs [13], offer global health electives. Our results show that a majority of responding anesthesiology residency PDs, even those that do not offer electives, agree that global health training is important and that exposure to international healthcare efforts in underserved regions is valuable. Of the respondents without global health opportunities, almost half were interested in initiating such electives. Beyond our primary aim, our exploratory analysis of attitudes towards global health electives revealed significant differences between those with existing programs and those without. While no causation can be inferred, one possibility is that this reflects that PD attitudes drive the establishment of a global health elective, another is that the existence of global health electives influences the attitudes of PDs, and a third is that these globally oriented PDs self-selected into culturally similar employment positions that match their values and are primed to invest in resident education.

With appropriate preparation, global health experiences for post-graduates have the potential to meet each of the six core competencies of the ACGME [14, 15]. Our study demonstrated that while 77% of responding programs with electives had established educational goals, training was neither comprehensive nor systematic. For example, beyond clinical preparation some programs provided curricula based on relevant biosocial determinants of health, as identified by the United Nations [16, 17] (e.g. poverty, gender violence, and access to natural resources), while others provided little foundational understanding in these arenas. Additionally, while the importance of sustained participation on the ground has been well documented [18], anesthesiology residents participated in electives for a median of just 10 days. Research was found to be only a minor component of the surveyed initiatives at 18% (*n* = 6), similar to general surgery (11%) and emergency medicine programs (26%); this is not surprising, as elective-based programs are twice as prevalent as research-based programs among offerings in internal medicine, pediatrics, obstetrics and gynecology, family medicine, and psychiatry. Unlike other core areas of anesthesiology training, there exists no guiding academic authority on global health education and research, and no central, reliable source of educational materials. These factors may lead to programs teaching global health inconsistently, without the input of experts, resulting in an incomplete education of residents at best, and harmful or irresponsible medical practice at worst. Without appropriate education, visiting trainees may fail to deliver care that meets standards, disrupt the practice of local healthcare providers or existing healthcare infrastructure [19], inappropriately utilize scarce resources [20], or experience increased personal or professional stress [21] .

There are two important limitations to this study. The first is the susceptibility of the survey methodology to nonresponse selection bias, as attitudes toward global health and the existence of a global health elective could be related to the decision to respond to the survey. We performed analyses using two techniques for estimates of bias (one statistically derived, and the other empirically derived from prior research on response behavior), which taken together suggest that the absolute bias for the estimate of programs was within 6.5%, which would not alter the qualitative conclusions. Web-based surveys are at risk for low response rates [22], although our rate of 44% was comparable to response rates in similar web-based survey studies on global health conducted by other medical specialties such as general surgery (29%) [13], obstetrics and gynecology (28%) [23], and emergency medicine (53%) [20]. The second important limitation is that the survey instrument we used, although informed by prior research, is novel and has not undergone validation analysis. The questions and results are represented as being clustered into multiple constructs, but it must be noted that these constructs were conceptually derived, and have not been assessed in exploratory or confirmatory factor analysis.

## Conclusions

The data from this study demonstrate that global health opportunities in anesthesiology, though similar to offerings in other medical specialties, is not readily accessible, comprehensive, or consistent. There is significant variation in program structure, goals, curriculum, and funding. Attitudes of PDs differ between programs with and without electives, which may reflect bidirectional influence to be investigated further. Future investigations may aim to identify milestones in global health literacy and incorporate standardized competencies in systematic education, with the guidance of experts in the field. The data from this study are critically relevant to educators who are designing global health programs for trainees. They highlight opportunities to improve education in the field of global anesthesiology and describe the critical starting points.

## Additional files

Additional file 1: Program Director Questionnaire. (PDF 77 kb)

Additional file 2: Comprehensive survey results. (PDF 122 kb)

## Abbreviations

ACGME: Accreditation Council for Graduate Medical Education
IRB: Institutional Review Board
MCAR: Missing completely at random
MNAR: Missing not at random
PD: Program Director
STROBE: Strengthening the Reporting of Observational studies in Epidemiology

## Footnotes

1
https://apps.acgme.org/ads/Public/Reports/Report/1. Last accessed October 12, 2016
