# Supplementary material for: Global health education in United States anesthesiology residency programs: a survey of resident opportunities and program director attitudes
Source: BMC Med Educ. 2017 Nov 16;17:215. doi: 10.1186/s12909-017-1056-3 (PMC5689206; doi:10.1186/s12909-017-1056-3)
Supplement: Supplementary file 1 — Program Director Questionnaire. (PDF 77 kb) [file 12909_2017_1056_MOESM1_ESM.pdf]

Appendix 1. Program Director Questionnaire

1) Does your anesthesiology residency training program offer a global health elective (an elective training opportunity outside of the United States)?

Yes
No

If Yes to question 1, skip to question 4, "How many residents per year are allowed to participate in a global health elective?" If No, proceed to question 2.

2) Would you like for your residency training program to offer a global health elective?

Yes
No
Unsure

If No, skip to question 19. If Yes or Unsure, proceed to question 3.

3) Please select any of the options listed below that may describe why your program does not offer global health elective opportunities (select all that apply).

A. Lack of interest in global health electives among residents
B. Lack of perceived volume in global health electives for residents
C. Lack of funding
D. Lack of time within parameters of training
E. Lack of global health partner or program through which to offer an elective
F. Other (specific):______

Anyone answering question 3 should now skip to question 19.

4) How many residents per year are allowed to participate in a global health elective?

___# Residents

5) Which residents, in terms of postgraduate year, are eligible to complete a global health elective? Check all that apply.

A. PGY1
B. PGY2
C. PGY3
D. PGY4

6) Approximately how long is the duration of the global health elective available through your training program?

___# Days
___# Weeks
___# Months

7) How many weeks of the elective, if any, is the resident required to use as vacation time?

A. None
B. 1 Week
C. 2 Weeks
D. 3 Weeks
E. 4 Weeks

8) With what frequency are residents in your training program allowed to participate in global health electives?

A. Yearly
B. Semi-annually
C. Only once during their entire residency

Appendix 1. Program Director Questionnaire

9) Does your elective address any of the following biosocial factors impacting health? Please check all that apply.

A. Poverty
B. Access to natural resources
C. Discrimination
D. Gender violence
E. Other (specify):______

If you checked any of the options above, please elaborate on how you address these topics in your elective.

10) To which country outside the US are your residents able to complete a global health elective? Please list all relevant countries.

11) Does your department utilize the help of a partner in locations where you offer global health electives? If yes, please select all that apply. If no, please select option C.

A. Yes, we have an educational institution partner through which we offer a global health elective (ex. International medical school, etc)
B. Yes, we have a nongovernmental organization partner through which we offer a global health elective
C. No, we do not have a partner

12) Funding for global health electives offered through your program is accomplished by (select the single source that provides the majority of funds if more than one source exists)

A. Internal department funding or endowment
B. Institutional funding
C. Nongovernmental organization funding
D. Grant funding not from a nongovernmental organization
E. None, residents must find funding on their own
F. Other (please describe):______

13) Does your department have established educational outcome goals for each resident while away on a global health elective?

Yes
No

14) Is there currently a research component required as part of the global health elective?

Yes
No

15) How are residents evaluated on their global health elective (select all that apply)? If they are not evaluated, please select H.

A. Intradepartmental attending anesthesiologist(s) evaluate residents
B. Local (international) attending anesthesiologist(s) evaluate
C. Didactic examination
D. Oral examination
E. Presentation
F. Requirement checklist (ex. Type and quantity of procedures or consultations performed)
G. Research project (paper or poster presentation)
H. We do not evaluate residents on their global health elective
I. Other (specify):______

16) Does a program faculty member(s) attend the global health elective with his or her residents?

Yes
No

Appendix 1. Program Director Questionnaire

17) Does at least one of your faculty members have global volunteer experience?
 Yes
 No

18) Does your program financially support residents seeking non program-sponsored mission trips abroad?
 Yes
 No

19) Please indicate the amount to which you agree with the following statements: Strongly agree, Somewhat agree, Somewhat disagree, Strongly disagree, No opinion
 A. Global health electives are important in the training of anesthesiology residents
 B. Exposure to global health care is a valuable experience for anesthesiology residents
 C. Exposure to global health care should be required of anesthesiology residency training

20) I believe that the benefits of global health electives include (select all that apply):
 A. Advancing education in the field of global anesthesia
 B. Generating effective and engaging programs in the developing world that give my department's residents and faculty the opportunity to become well-rounded, globally conscious physicians
 C. The opportunity for residents to become leaders in the field of global anesthesia research and contribute to global health literature
 D. Developing cross-institutional collaborations
 E. Personal, professional, and institutional development in the spheres of service-oriented action, humanitarian contribution, and outreach to underprivileged individuals and societies
 F. Providing needed health care to underserved area of developing countries
 G. Enhancing resident training through exposure to pathological conditions less commonly encountered during training in the United States
 H. Enhancing resident training through exposure to foreign physicians
 I. I do not believe that there are any benefits to global health electives
 J. Other (please specify):______
