# Supplementary material for: Global health education in United States anesthesiology residency programs: a survey of resident opportunities and program director attitudes
Source: BMC Med Educ. 2017 Nov 16;17:215. doi: 10.1186/s12909-017-1056-3 (PMC5689206; doi:10.1186/s12909-017-1056-3)
Supplement: Supplementary file 2 — Comprehensive survey results. (PDF 122 kb) [file 12909_2017_1056_MOESM2_ESM.pdf]

Appendix 2. Comprehensive survey results.

Sample without global health elective	Total N=22
**Would like residency training to have global health elective, no. (%)**	10 (45.5)
**Reasons for lack of global health elective, no. (%)**	
Lack of interest	3 (13.6)
Lack of perceived volume of cases abroad	0 (0)
Lack of funding	13 (59.1)
Lack of time within parameters of training	8 (36.4)
Lack of global health partner/program through which to offer an elective	11 (50.0)

Sample with global health electives	Total N=34
**Partners, no. (%)**	**N=32**
Internal department funding or endowment	12 (37.5)
Institutional funding	4 (12.5)
Nongovernmental organization funding	5 (15.6)
Grant funding not from a nongovernmental organization	3 (9.4)
Residents must find their own funding	1 (3.1)
**Curriculum includes biosocial determinants of health, no. (%)**	**N=34**
Contains content on poverty	23 (67.6)
Contains content on access to natural resources	14 (41.2)
Contains content on discrimination	9 (26.5)
Contains information on gender violence	3 (8.8)
**Goals of global health elective, no. (%)**	**N=34**
Programs with educational outcome goals	26 (76.5)
Programs with required research component	6 (17.6)
**Elective characteristics, no. (%)**	**N=34**
Duration of elective in days, median (interquartile range)	10 (5-30)
Reported discrete number of residents per year allowed to participate, minimum-maximum	1-18
**Resident eligibility to participate, no. (%)**	**N=34**
PGY 1	0 (0.0)
PGY 2	4 (11.8)
PGY 3	24 (70.6)
PGY 4	32 (94.1)

Requirement that elective be used as vacation time, no. (%)	N=33
Yes	1 (3%)
No	32 (97%)

Frequency with which residents are allowed to participate, no. (%)	N=34
Yearly	11 (32.4)
Semi-annually	3 (8.8)
Only once during their entire residency	20 (58.8)

How residents are evaluated, no. (%)	N=31
By intradepartmental attending anesthesiologists	19 (61.3)
By local (international) attending anesthesiologists	6 (19.4)
Presentation	1 (3.2)
Research Project	1 (3.2)
No evaluation	1 (3.2)
Other	3 (9.7)

Program countries, no. (%)	N=33
Dominican Republic	6 (18.2)
Ethiopia	6 (18.2)
India	6 (18.2)
China	5 (15.2)
Ecuador	5 (15.2)
Ghana	4 (12.1)
Guatemala	4 (12.1)
Kenya	4 (12.1)
Haiti	3 (9.1)
Honduras	3 (9.1)
Malawi	3 (9.1)
Nicaragua	3 (9.1)
Peru	3 (9.1)
Philippines	3 (9.1)
Tanzania	3 (9.1)
Colombia	2 (6.1)
Mexico	2 (6.1)
Rwanda	2 (6.1)
South Africa	2 (6.1)
Botswana	1 (3.0)
El Salvador	1 (3.0)
Fiji	1 (3.0)
Guyana	1 (3.0)
Jamaica	1 (3.0)
Liberia	1 (3.0)
Madagascar	1 (3.0)
Mozambique	1 (3.0)
New Zealand	1 (3.0)
Niger	1 (3.0)

Uganda	1 (3.0)
Ukraine	1 (3.0)
Vietnam	1 (3.0)
Zambia	1 (3.0)
**Local partners, no. (%)**	**N=32**
Yes, we have an educational institution partner through which we offer a global health elective	9 (28.1)
Yes, we have a non-governmental organization partner through which we offer a global health elective	12 (37.5)
No, we do not have a partner	11 (34.4)
**Faculty, no. (%)**	**N=31**
Faculty member in attendance	26 (83.9)
At least one faculty member with global experience	31 (100.0)
**Offer financial support to residents seeking non-program-sponsored trips abroad, no. (%)**	**N=30**
Yes	10 (33.3)
No	20 (66.7)

Sample characteristics	With program N=31	Without program N=19	Total N=50	P-value
**Value of global health in anesthesiology residency, no. (% indicating strongly or somewhat agree)**				
Electives are important in training of anesthesiology residents	27 (87.1)	8 (42.1)	35 (70.0)	< 0.001
Exposure to global health care is a valuable experience	29 (93.5)	13 (68.4)	42 (84.0)	0.02
Exposure to global health care should be required of anesthesiology residency training	10 (32.3)	1 (5.3)	11 (22.0)	0.03
**Perceived benefits of global health electives, no. (%)**				
Advancing education in the field of global anesthesia	28 (90.3)	8 (42.1)	36 (72.0)	< 0.001
Generating effective and engaging programs in the developing world that give my department 's residents and faculty the opportunity to become well-rounded, globally conscious physicians	23 (74.2)	8 (42.1)	31 (62.0)	0.02
The opportunity for residents to become leaders in the field of global anesthesia research and contribute to global health literature	21 (67.7)	6 (31.6)	27 (54.0)	0.01
Developing cross-institutional collaborations	21 (67.7)	6 (31.6)	27 (54.0)	0.01
Personal, professional, and institutional development in the spheres of service-oriented action, humanitarian contribution, and outreach to underprivileged individuals and societies	25 (80.6)	14 (73.7)	39 (78.0)	0.56
Providing needed health care to underserved area of developing countries	24 (77.4)	15 (78.9)	39 (78.0)	0.90
